# Bridging Data Science to Policy: Introducing an *HDS* Environmental Health Special Issue

**DOI:** 10.34133/hds.0516

**Published:** 2026-09-18

**Authors:** Qimin Zhan

**Affiliations:** Editor-in-Chief, *Health Data Science*.

Global environmental change has become a consequential threat to human health. Climate change, air pollution, extreme weather, water insecurity, and persistent chemical contamination are altering patterns of illness, disability, and premature death [1,2]. These hazards are pervasive but not evenly shared. Countries and communities with fewer resources for monitoring, health care, infrastructure, and adaptation may face substantial exposure despite having contributed less to global emissions, while vulnerable groups within all settings remain at risk [1,3]. Older adults and people living with cardiovascular, respiratory, kidney, or metabolic disease deserve particular attention because environmental stress can interact with frailty, comorbidity, and limited capacity to respond [4,5]. Environmental health has therefore become a test of whether health systems can recognize upstream risks early enough, and act fairly enough, to prevent avoidable harms.

This challenge motivated the special issue Health Impacts of Global Environmental Changes: Bridging Data Science to Policy. Its aim is not to provide a comprehensive environmental science review but to offer scientifically and quantitatively grounded contributions asking how evidence can inform policies and actions to protect health in a changing global environment. Recent work on lung cancer illustrates this evidence-to-policy pathway by quantifying how coordinated clean-air and carbon-neutrality policies could sustain reductions in disease burden in a rapidly aging population [6]. The collection also responds to a wider international agenda: The COP28 UAE Declaration on Climate and Health called for climate-resilient, sustainable, and equitable health systems and for stronger capacity to protect vulnerable populations from climate-related health risks [7]. *Health Data Science* is a suitable platform for this conversation because its mission of *Data for Better Health* and its scope—from data resources and governance to analytical innovation, clinical and population applications, and translation into health benefits—enable it to connect evidence on environmental exposures and health impacts with action [8,9]. Authored by environmental health scientists, clinicians, and public health researchers from leading institutions in China and abroad, these articles reflect a shared agenda that depends on sustained cross-disciplinary collaboration and continued advocacy across research, policy, and practice.

The articles currently included in the special issue approach this agenda through three connected questions. First, how can data make unequal environmental-health burdens, accountability, and development-stage differences visible rather than reducing them to global averages? Second, once risks are identified, how can measurements, standards, and early-warning thresholds turn environmental information into actionable signals for prevention and policymaking? Third, how can such evidence support preparedness for organ-specific vulnerability and the needs of vulnerable populations? The sequence is moving from comparative burden to warning systems, exposure governance, and health management.

The opening problem is fairness in the production and distribution of environmental health risk. Global burden estimates can quantify how health loss is distributed across populations and places, but may obscure how development pathways and emissions generate hazards, how demographic change shapes vulnerability, and how unequal measurement capacity determines which burdens become visible. Zhou and colleagues examined 2000–2019 country-level mortality attributable to landscape-fire-sourced air pollution and floods, linking these estimates to country development and per capita carbon dioxide emissions [10]. They found that the heaviest burdens fell disproportionately on the lowest-emitting countries. Mortality from landscape-fire-related air pollution was more strongly associated with socioeconomic development and showed a steeper gradient than flood-related mortality. By placing inequality, accountability, and justice within a unified climate-health governance framework, the article makes a thoughtful and valuable contribution to a more equitable climate-health agenda.

Ambient air pollution remains one of the foremost environmental contributors to premature death worldwide. Jiang and colleagues [11] used Global Burden of Disease 2021 estimates to examine regional trends in mortality attributable to ambient particulate matter (PM2.5) and ozone from 1990 to 2020. They identified a development trap in regions with a middle sociodemographic index, where rapid industrialization and an epidemiological shift toward pollution-sensitive noncommunicable diseases combine to amplify population health risks. This environmental Kuznets curve-type pattern informs stage-specific health policies that reduce both exposure and population vulnerability. Its development-sensitive analysis is a valuable contribution to making air-pollution policy more targeted and effective.

A second question is how environmental health risks should be measured and translated into standards and early-warning thresholds that prompt timely action. Li and colleagues [12] focus on high-impact pollution weather, arguing that long-term averages and conventional air quality index categories may understate short-lived PM2.5 spikes that can trigger cardiovascular harm. Their proof-of-concept combines a recently developed function relating short-term PM2.5 exposure to cardiovascular mortality with exposure, population, and mortality data from China to assess trade-offs between estimated avoided deaths and alert burden. Their analysis illustrates a paradox: As air quality standards become more stringent, unchanged warning thresholds may leave some unsafe exposures insufficiently covered. The article should therefore be read as a framework for health-oriented validation of risk-based nowcasting, not a definitive national threshold. Importantly, this study offers a valuable bridge from short-term pollution dynamics to actionable public-health protection.

Chronic-disease prevention faces a related visibility problem: Exposures to persistent chemicals are not routinely captured in disease-burden assessments or incorporated into policy evaluation. Gao and colleagues [13] address what they term the exposure dimension gap, using per- and polyfluoroalkyl substances and diabetic kidney disease as an illustrative case. Their Perspective connects biomonitoring, environmental monitoring, disease registries, exposure-response evidence, burden estimation, and policy evaluation within an exposure–burden–policy cycle. It asks how chronic-disease prevention might incorporate environmental exposures in a way that can be evaluated over time. The Perspective positions environmental exposures as a measurable and policy-relevant dimension of chronic disease prevention.

The third question is how evidence from population surveillance can inform clinical preparedness. The kidney is a sentinel organ for environmental stress, and F. Zhang and L. Zhang [14] propose a data-to-policy roadmap that considers how linked environmental and health data, causal-inference approaches, artificial intelligence, risk prediction, forecasting, and genetic risk analysis could support long-term mitigation and short-term adaptation. This Perspective provides an example applicable to other environmentally influenced diseases, raising the diagnosis and treatment of disease from a clinical concern into an integrated instrument of climate governance. The roadmap provides a structured pathway for translating evidence on environmental risks to kidney health into policy and clinical implementation.

Preparedness must account for older people’s needs and ensure that adaptation systems are accessible to them. Wang and Xue [15] turn the discussion to population aging and later-life resilience. Their proposed “Green Longevity” framework connects cleaner environments with resilient health services, age-friendly communities, accessible warnings, green travel, and social participation. Such synergistic solutions operationalize the One Health concept and respond to the paradigm shift required to meet the global Sustainable Development Goals. The proposed framework integrates healthy aging into climate adaptation through coordinated environmental, health-service, and community strategies.

The contributions to this special issue share a practical goal: to use evidence to identify unequal health burdens, inform standards and surveillance, and guide adaptation that reaches populations at greatest risk. *Health Data Science* invites environmental health researchers, clinicians, data scientists, engineers, ethicists, and policymakers to develop pathways from data to action that are scientifically rigorous and usable in practice. On a changing planet, protecting human health and the environment must proceed together.
